# Combined modified atmosphere packaging and low temperature storage delay lignification and improve the defense response of minimally processed water bamboo shoot

**DOI:** 10.1186/1752-153X-7-147

**Published:** 2013-09-04

**Authors:** Lili Song, Hangjun Chen, Haiyan Gao, Xiangjun Fang, Honglei Mu, Ya Yuan, Qian Yang, Yueming Jiang

**Affiliations:** 1 Food Science Institute, Zhejiang Academy of Agricultural Sciences, Hangzhou 310021, The People’s Republic of China; 2 South China Botanical Garden, The Chinese Academy of Sciences, Guangzhou, LeYiJu 510650, The People’s Republic of China

**Keywords:** Water bamboo shoot, Modified atmosphere packaging, Lignification, Antioxidant enzyme, Membrane integrity, Energy

## Abstract

**Background:**

Minimally processed water bamboo shoot (WBS) lignifies and deteriorates rapidly at room temperature, which limits greatly its marketability. This study was to investigate the effect of modified atmosphere packaging (MAP) on the sensory quality index, lignin formation, production of radical oxygen species (ROS) and activities of scavenging enzymes, membrane integrity and energy status of minimally processed WBS when packaged with or without the sealed low-density polyethylene (LDPE) bags, and then stored at 20°C for 9 days or 2°C for 60 days.

**Results:**

The sensory quality of minimally processed WBS decreased quickly after 6 days of storage at 20°C. Low temperature storage maintained a higher sensory quality index within the first 30 days, but exhibited higher contents of lignin and hydrogen peroxide (H_2_O_2_) as compared with non-MAP shoots at 20°C. Combined MAP and low temperature storage not only maintained good sensory quality after 30 days, but also reduced significantly the increases in lignin content, superoxide anion ($O_{2}^{.-}$) production rate, H_2_O_2_ content and membrane permeability, maintained high activities of superoxide dismutase (SOD), catalase (CAT) and ascorbate peroxidase (APX), and reduced the increase in activities of lipase, phospholipase D (PLD) and lipoxygenase (LOX). Furthermore, the minimally processed WBS under MAP condition exhibited higher energy charge (EC) and lower adenosine monophosphate (AMP) content by the end of storage (60 days) at 2°C than those without MAP or stored for 9 days at 20°C.

**Conclusion:**

These results indicated that MAP in combination with low temperature storage reduced lignification of minimally processed WBS, which was closely associated with maintenance of energy status and enhanced activities of antioxidant enzymes, as well as reduced alleviation of membrane damage caused by ROS.

## Background

Water bamboo shoot (WBS, *Zizania aquatica* L.) is a perennial aquatic vegetable with high nutritional and commercial values, originating from south China. The outer leaf sheaths from water bamboo shoot are usually removed, which is considered a minimally processing. The minimally processed WBS has become increasingly popular in recent years because of its freshness and convenience for consumers, but the minimally processed WBS deteriorates rapidly and results in lignification and decay development under ambient temperature condition [1]. Lignification of the minimally processed WBS is a major problem that influences greatly quality and limits its marketability.

Biosynthesis of lignin is a complex process and regulated usually by radical oxygen species (ROS). Among them, hydrogen peroxide (H_2_O_2_) induces polymerization of different subunits of lignin (4-hydroxy cinnamyl aldehyde, coniferyl alcohol and sinapoyl alcohol), leading to a complete lignin deposition [2]. Recent research indicates that the enhanced lignification of plant tissues by ROS is due to the imbalance between ROS and ROS-scavenging systems during senescence or under stress conditions [3,4]. It has been suggested that a loss in antioxidant capacity in plant tissues results in an intrinsic accumulation of H_2_O_2_, which could then act as a signalling molecule triggering lignification [5]. In addition, membrane phospholipids are the major targets for ROS which can mediate membrane damage, lipid peroxidation and increase membrane permeability [6]. It has been reported that there were obvious increases in membrane permeability and malondialdehyde (MDA) content accompanied by accumulation of lignin in button mushrooms [7].

Energy plays a pivotal role in maintenance of membrane integrity and lipid metabolism. The depletion of adenosine triphosphate (ATP) leads to membrane damage and reduces lipid synthesis [8]. Rawyler et al. [9] demonstrated that a threshold existed in ATP production rate, below which membrane lipids started to hydrolyze in potato cells. Recent studies suggest that not only increased membrane permeability but also enhanced ROS production is closely associated with low levels of ATP production and energy charge (EC) in harvested horticultural crops [10]. Kibinza et al. [11] demonstrated that both lipid oxidation and ATP depletion induced by H_2_O_2_ were attributed to sunflower seed deterioration. Thus, high energy status could maintain membrane function or reduce ROS production via ROS scavenging enzymes and non-enzyme antioxidants, thereby, delaying ripening or senescence of horticultural crops [12-14].

Modified atmosphere packaging (MAP) can extend shelf-life and maintain the quality of many intact and fresh-cut horticultural crops through creating modified atmosphere in packages [15]. This treatment could lead to off-flavor or flavor loss as well as quality deterioration due to impropriate temperature storage. Evidence has shown that combined MAP treatment and low temperature storage can overcome well the problem [16,17]. Xie et al. [18] found that intact WBS stored in MAP exhibited low levels of lignin content and lignified-related enzyme activity at 1°C. Unfortunately, there is little published data on effect of the combined MAP and low temperature storage on minimally processed WBS. Additionally, it is still unclear what the mode of action improves energy status, membrane integrity and activities of antioxidant enzymes.

The objective of this work was to investigate the sensory quality, lignin formation and ROS production and the changes in activities of superoxide dismutase (SOD), catalase (CAT), ascorbate peroxidase (APX), lipase, phospholipase D (PLD) and lipoxygenase (LOX) and energy status in minimally processed WBS stored in MAP at room and low temperatures.

## Results

### Changes in gas concentrations

**Figure 1:**
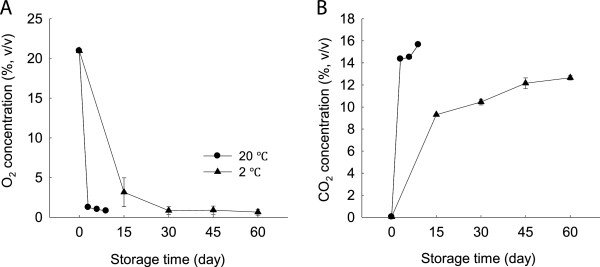
**O**_**2 **_**and CO**_**2 **_**concentrations in modified atmosphere package in minimally processed WBS during storage at 20 and 2°C. A**-O_2_; **B**-CO_2_. Data were average values ± standard errors (n=3).

As shown in Figure 1, O_2_ concentration in MAP condition decreased sharply within the first 3 days and then continued to decrease at a much lower level at 20 or 2°C. In contrast, CO_2_ concentration in MAP increased rapidly within the first 3 days at 20°C and 15 days at 2°C, respectively, and then continued to increase slowly. After 9 days of storage at 20°C, CO_2_ concentration reached 15.7%. However, even after 60 days at 2°C, CO_2_ concentration remained comparatively low and only reached 12.6%. Thus, it indicated clearly that the minimally processed WBS stored in MAP accumulated CO_2_ more slowly as compared with those without MAP at higher temperature.

### Changes in sensory quality and lignin content

**Figure 2:**
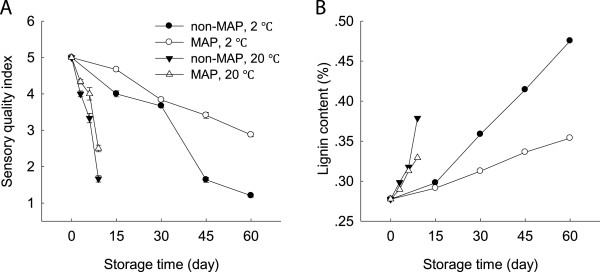
**Effect of MAP on sensory quality index and lignin content of minimally processed water bamboo shoot during storage at 20 and 2°C. A**-sensory quality index; **B**-lignin content. Data were average values ± standard errors (n=3).

The sensory quality of the minimally processed WBS decreased quickly. The minimally processed WBS showed an obvious green and yellow appearance with substantial water loss after 6 days at 20°C. Low temperature storage kept good sensory quality of the minimally processed WBS within the first 30 days. After 30 days of storage at 2°C, the sensory quality decreased quickly. The minimally processed WBS stored in MAP at 2°C kept better sensory quality during the late stage of storage, with the shoot tissues remaining relatively firm and plump with only slight water loss after 60 days of storage (Figure 2A).

The lignin content of the minimally processed WBS increased by 37% after 9 days as compared that of 0 day at 20°C, which was coincidence with the decrease of sensory quality (Figure 2). The shoots stored at ambient temperature lost their sensory quality after 9 days, which was not acceptable for consumer. It was interesting that the minimally processed WBS stored at 2°C increased lignin content after storage of 60 days compared to those stored at 20°C for 9 days. MAP reduced the increase in lignin content, with the lignin content of the minimally processed WBS stored in MAP being about 74% of those without MAP after 60 days at 2°C (Figure 2B).

### Changes in $O_{2}^{.-}$ production rate and H_2_O_2_ content

**Figure 3:**
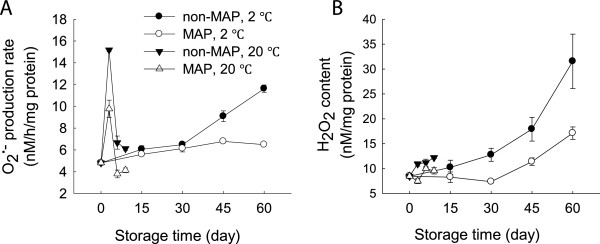
**Effect of MAP on **$O_{2}^{.-}$**production rate and H**_**2**_**O**_**2 **_**content of minimally processed water bamboo shoot during storage at 20 and 2°C. A**-$O_{2}^{.-}$ production rate; **B**-H_2_O_2_ content. Data were average values ± standard errors (n=3).

At 20°C, $O_{2}^{.-}$ production rate increased within the first 3 days, and then decreased thereafter. MAP reduced the $O_{2}^{.-}$ production rate but no significant (p ≥ 0.05) difference existed between the MAP- and non-MAP-treated WBS in the storage period at room temperature (Figure 3A). In contrast, $O_{2}^{.-}$ production rate of the minimally processed WBS steadily increased over time at 2°C while MAP markedly reduced the $O_{2}^{.-}$ production rate. The $O_{2}^{.-}$ production rate of the minimally processed WBS stored in MAP was 56% of those without MAP after 60 days at 2°C (Figure 3A).

H_2_O_2_ content exhibited a very different pattern compared to the $O_{2}^{.-}$ production rate. At two storage temperatures, H_2_O_2_ content increased steadily over time and the minimally processed WBS exhibited higher levels of H_2_O_2_ after 60 days than those stored at room temperature for 9 days. MAP decreased H_2_O_2_ level of the minimally at low temperature processed WBS in this storage period at both temperatures, with significant (p ≤ 0.05) difference between MAP and non-MAP after 60 days (Figure 3B).

### Changes in activities of SOD, CAT and APX

**Figure 4:**
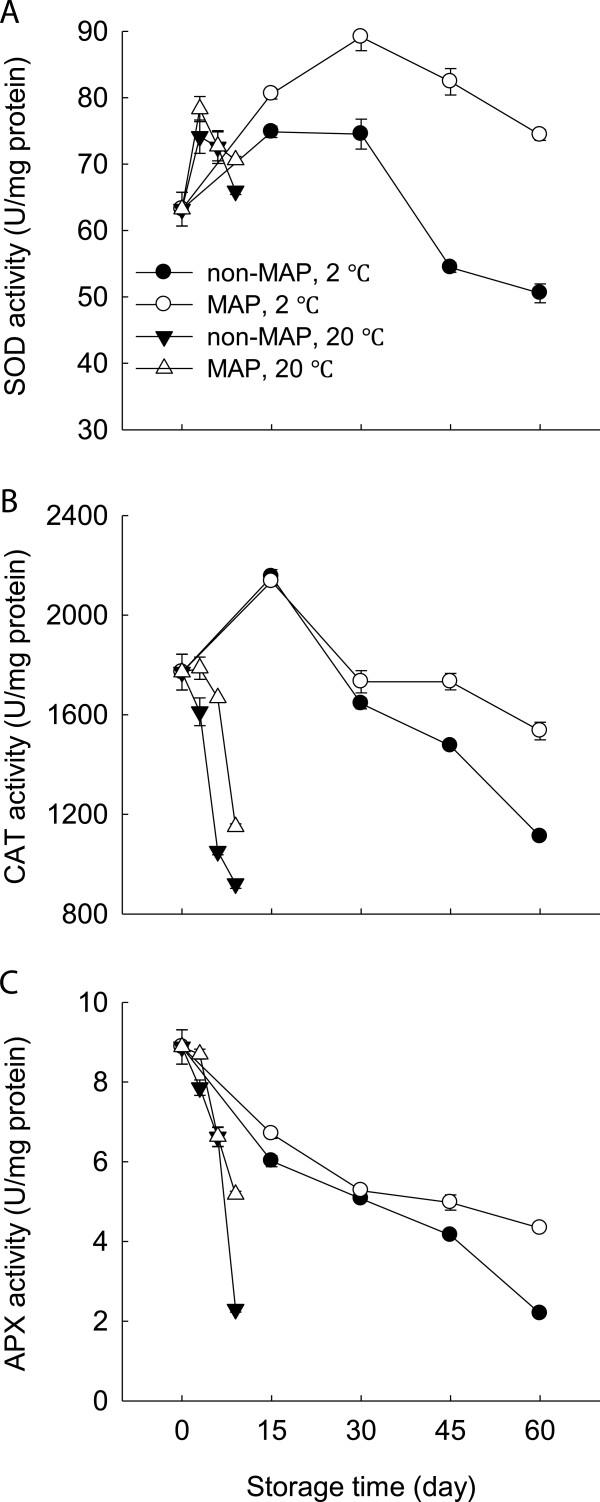
**Effect of MAP on activities of SOD, CAT and APX of minimally processed water bamboo shoot during storage at 20 and 2°C. A**-SOD; **B**-CAT; **C**-APX. Data were average values ± standard errors (n=3).

Figure 4 presents the activities of SOD, CAT and APX in the minimally processed WBS at two storage temperatures. SOD activity increased within the first 6 days at 20°C and 15 days at 2°C, respectively, and then decreased steadily. The activities of CAT and APX were observed to decrease during storage, whilst the decreases occurred much more rapidly at 20°C. At 20°C, there were similar profiles of activities of SOD, CAT and APX in the MAP and non-MAP minimally processed WBS, but no significant (p ≥ 0.05) difference existed. MAP delayed significantly (p ≤ 0.05) the decrease in activities of SOD, CAT and APX of the minimally processed WBS after 30 days at 2°C.

### Changes in membrane permeability and activities of PLD, lipase and LOX

**Figure 5:**
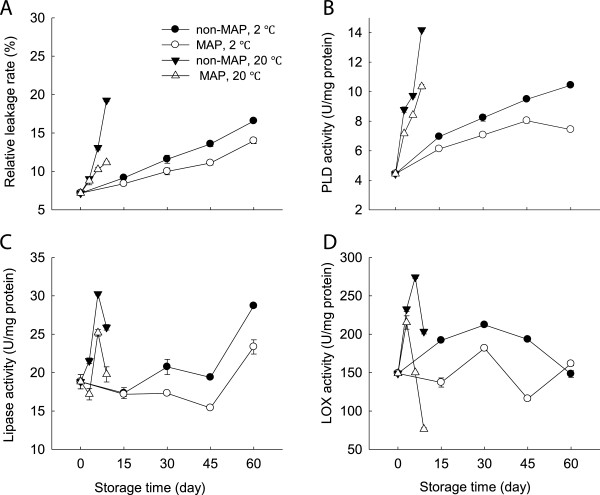
**Effect of MAP on electrolyte leakage rate and activities of PLD, lipase and LOX of minimally processed water bamboo shoot during storage at 20 and 2°C. A**-electrolyte leakage rate; **B**-PLD; **C**-lipase; **D**-LOX. Data were average values ± standard errors (n=3).

Membrane permeability of the minimally processed WBS increased as time progressed and reached a maximum of 19.2% after 9 days at 20°C, but only 16.6% after 60 days at 2°C (Figure 5A). The minimally processed WBS stored in MAP had relatively low leakage rate compared with those without MAP after 60 days at 2°C (Figure 5A).

PLD activity was observed to increase quickly for the minimally processed WBS stored at 20°C, with a maximum after 9 days, but the enzymatic activity after 60 days at 2°C was much lower than those stored for 9 days at 20°C. MAP reduced the further increase in PLD activity of the minimally processed WBS at 2°C (Figure 5B).

Lipase and LOX activities had similar profiles at 20°C, which increased rapidly within the first 6 days and then decreased quickly (Figure 5C and D). MAP reduced the increases in activities of lipase and LOX, but no significant differences between MAP and non-MAP were observed (p ≥ 0.05) (Figure 5C and D). In contrast, at 2°C, lipase activity increased over time and LOX activity increased within the first 30 days and then decreased, while MAP reduced the increases in lipase and LOX activities (Figure 5C and D).

### Changes in contents of ATP, ADP and AMP and EC

**Figure 6:**
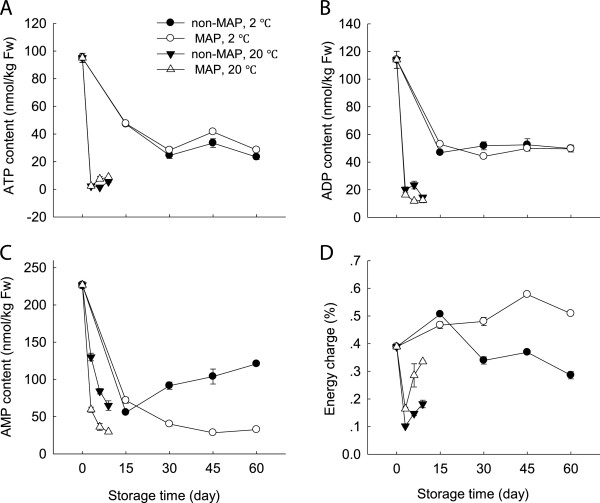
**Effect of MAP on contents of ATP, ADP and AMP and EC value of minimally processed water bamboo shoot during storage at 20 and 2°C. A**-ATP; **B**-ADP; **C**-AMP; **D**-EC value. Data were average values ± standard errors (n=3).

It was found that the minimally processed WBS had high contents of ATP, ADP and AMP but a low EC level before storage. As storage time progressed, the levels of ATP and ADP decreased (Figure 6A and B). This decrease was more apparent at room temperature compared to low temperature, with a much lower level after 9 days at 20°C than after 60 days at 2°C. However, the changes in contents of ATP and ADP between the MAP and non-MAP WBS were not significant (p ≥ 0.05) at both storage conditions (Figure 6A and B). Interesting, AMP content of the minimally processed WBS started to accumulate at a steady rate after 15 days, but under MAP condition the AMP content continued to decrease over time at 2°C (Figure 6C).

It was interestingly noted the changes in EC profiles of the minimally processed WBS at low and room temperatures (Figure 6D). At 20°C, EC reduced quickly within the first 3 days and then slowly increased, while at 2°C, EC increased within the first 15 days and then decreased steadily over time. Application of MAP at 2°C inhibited this decrease in the EC level.

## Discussion

Minimally processed WBS lignifies and deteriorates rapidly during room temperature storage, which is characterized by lignin accumulation and rot development (Song et al., 2011). Low temperature storage is considered to be an essential technology for delaying decay and quality loss in general. With storage progress, cold storage still leads to increased lignification and tissue toughening over time [19]. In this study, we also found that the minimally processed WBS stored at 2°C had higher lignin content after 60 days of storage than those stored at room temperature although cold storage maintained good sensory quality within the first 30 days (Figure 2). This result was consistent with results reported by Ding et al. [17]. Application of MAP can obtain better beneficial effect at lower storage temperatures by controlling the internal atmospheric conditions (Figure 1). Higher respiration and storage temperatures may lead to excessive accumulation of CO_2_ and/or depletion of O_2_ inside the packages [16]. These sorts of conditions are likely to lead to metabolic disorders and reduce produce quality. Our observations suggested that CO_2_ levels were indeed correlated with WBS lignifications after 3 days of storage at 20°C (R^2^ =0.98) and 15 days of storage at 2°C (R^2^ =0.96), respectively (Figure 1B and Figure 2B). Thus, the beneficial effects of MAP storage on the minimally processed WBS at low temperature might be due to low CO_2_ concentration inside the packages.

Lignification of harvested horticultural crops is closely associated with the overproduction of ROS which can cause oxidative damage to biomolecules [3,4,7]. ROS scavenging enzymes, such as SOD, CAT and APX, can protect plants from oxidative stress. SOD scavenges $O_{2}^{.-}$ into H_2_O_2_, which can be further converted into water by CAT and APX [20]. In this study, MAP-treated WBS exhibited lower levels of $O_{2}^{.-}$ production rate and H_2_O_2_ content and high activities of SOD, CAT and APX after 30 days of storage at 2°C compared to those without MAP (Figures 3 and 4). This result also indicated that MAP in combination with low temperature storage protected the minimally processed WBS from oxidative damage due to increased activities of SOD, CAT and APX. A positive correlation was observed between H_2_O_2_ level and lignin content of the minimally processed WBS stored at 2°C (R^2^ = 0.89). Thus, delayed lignifications in association with high activities of SOD, CAT, and APX were obtained when cold storage plus MAP was applied in this study. Similar result was described by Liu et al. [4], who found that sliced water bamboo shoots stored in polyethylene film bags had high levels of SOD and POD activities when stored at 2°C.

High levels of ROS implicate in lipid damage and alter membrane properties, resulting in an increase in membrane permeability [6]. PLD and lipase mediate lipid hydrolysis in membrane deterioration while LOX activates lipid peroxidation and involve in ripening of litchi fruit during cold storage [21]. Loss of membrane integrity may result in the release of these enzymes which could further promote degradation and peroxidation of membrane lipids [21]. As storage progressed, the relative leakage rate increased markedly at both 20 and 2°C, coinciding with increases in contents of H_2_O_2_ and lignin (Figures 2B, 3B and 5A), which suggested that ROS could increase membrane permeability leading to membrane damage. These observations were consistent with the report of Jiang et al. (2010) on button mushroom [7]. There were increases in activities of PLD, lipase and LOX accompanied by increased membrane permeability in the minimally processed WBS stored at 20 or 2°C (Figure 5). Application of MAP reduced the activities of these enzymes tested in this study. Membrane damage could be further delayed by the use of MAP in combination with low temperature storage in terms of these enzymatic activities (Figure 5B,C and D).

Evidence suggests that ROS production and membrane damage may be linked to a depletion of energy availability. A link between energy availability and membrane integrity is well established. For instance, Jiang et al. [10] have shown that loss of membrane integrity was likely to be due to limited energy availability during senescence and ripening of horticultural crops, while Kibinza et al. [11] have indicated that the accumulation of H_2_O_2_ might be due to ATP depletion during sunflower seed deterioration. Our results showed that as storage progressed, ATP, ADP and AMP concentrations of the minimally processed WBS decreased over time (Figure 6A,B and C). These observations were associated with increases in membrane permeability, $O_{2}^{.-}$ production, H_2_O_2_ content and WBS lignification (Figures 3, 5A and 6). Under the MAP and low temperature storage conditions, lower AMP content and higher EC level of the minimally processed WBS compared with those without MAP suggested that MAP combined with cold storage increased energy availability (Figure 6C and D).

**Figure 7:**
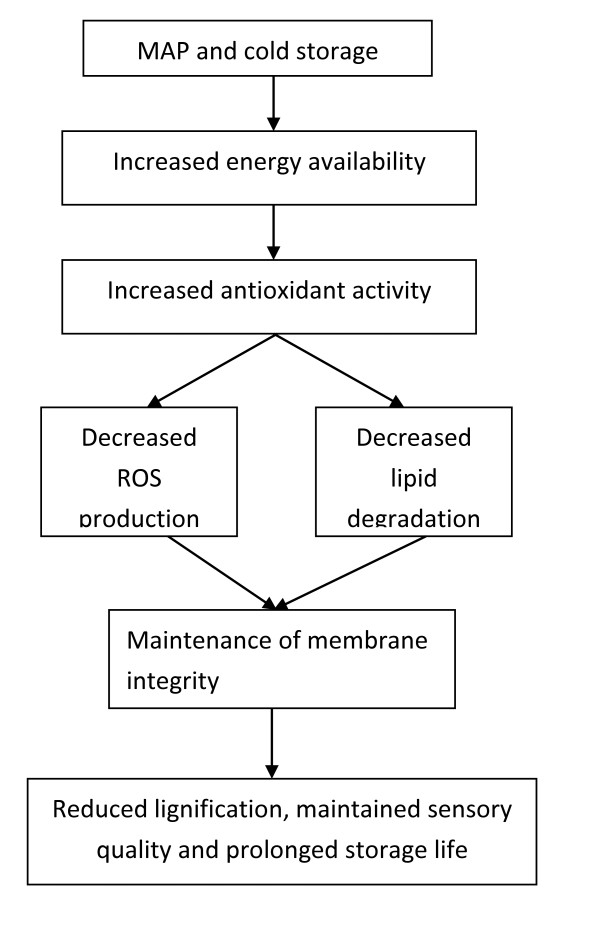
The hypothesis of the role of MAP combined with cold storage involved in reducing lignifications and increasing storage life of the minimally processed water bamboo shoot.

Given the findings presented in this study, a hypothesis to explain the role of MAP involved in reducing lignifications and increasing storage life of the minimally processed WBS was proposed in Figure 7. In this hypothesis, it was suggested that application of MAP and cold storage maintained energy availability, increased antioxidant activity, decreased ROS production and lipid degradation and maintained membrane integrity, which, in turn, reduced lignifications, maintained sensory quality and prolonged storage life of the minimally processed WBS.

## Materials and methods

### Plant materials

Fresh WBS *cv. Longjiao* No. 2, an autumn-cropping cultivar, was harvested from a plantation in Tongxiang, Zhejiang Province, China. The shoots were immediately transported to laboratory by car within 3 h and pre-cooled at 8–10°C overnight. WBS was selected for uniform shape, color and size and the absence of any blemishes or disease. The outer leaf sheaths were carefully peeled off by hand. About 5 cm was removed from the cut end of the shoot with a sharp knife.

### Packaging and storage temperature

The minimally processed WBS were placed into open or sealed low-density polyethylene (LDPE) bags (34.5 × 20.5 cm) and then stored for 9 days at 20°C and 90% relative humidity (RH) and for 60 days at 2°C and 90% RH, respectively. Five shoots were placed in each bag and 3 bags per treatment were used. LDPE bags were 0.05 mm in thickness with O_2_ and CO_2_ transmission rates of 1.2 × 10^-14^ M/m^2^/s/Pa and 10.8 × 10^-14^ M m^2^/s/Pa, respectively, detected with gas transmission rate tester at 25°C and 80% RH (BTY-B1, Labthink Instruments, Jinan, China). The minimally processed WBS prior to MAP application was identified as 0 day and the gas composition was not adjusted at the beginning of the storage period.

### Measurements of gas concentrations

A 1 ml gas sample was taken from the storage bag containing five shoots stored at 20 and 2°C for the measurements of gas concentrations. CO_2_ and O_2_ concentrations were determined using a portable gas analyzer (CYES-II, Shanghai Scientific Instruments, Shanghai, China).

### Sensory quality evaluation

Sensory quality was evaluated and scored using a modified 5 (excellent quality) to 1 (poor quality) scale after Song et al. [1]: where 5, fresh without any water loss (no shrinkage on appearance); 4, slight green appearance and water loss; 3, moderate green appearance and several water loss; 2, severe green appearance and water loss; 1, entirely green appearance and water loss. All shoots were scored until score 1 and the sensory quality index was calculated as Σ (sensory scale × percentage of corresponding shoot within each class).

### Lignin determination

Lignin was extracted and measured according to Liu et al. [3] with modifications. Frozen tissue powder (5 g) from 15 shoots were homogenized in 15 ml of 95% ethanol and subsequently centrifuged at 4°C for 10 min at 12000 × g. The pellet was washed three times for 15 min with 40 ml of ethanol and hexane (1:2, v/v) whilst being continuously stirred. After centrifugation at 12000 × g for 10 min, the final insoluble-alcohol lignin residue was determined gravimetrically by the method of Liu et al. [4]. Results were expressed as a g lignin per 100 g fresh weight (FW).

### Determination of membrane permeability

Membrane permeability, expressed as relative electrolyte leakage rate, was determined by the method of Jiang and Chen [22]. Relative leakage rate was expressed as a percentage of total electrolyte leakage.

### Determinations of $O_{2}^{.-}$ production rate and H_2_O_2_ content

Superoxide anion ($O_{2}^{.-}$) production rate was exacted from 4 g frozen tissue powder from 15 shoots and measured by monitoring the nitrite formation from hydroxylamine in the presence of $O_{2}^{.-}$ as described by Wang and Luo [23]. The $O_{2}^{.-}$ production rate was expressed as nM/h/mg protein.

For analysis of H_2_O_2_ content, 4 g of frozen tissue powder (15 shoots) were ground finely and homogenized with 20 ml of acetone at 0°C following the method of Patterson et al. [24]. H_2_O_2_ content was calculated using H_2_O_2_ as a standard and then expressed as nM/mg protein.

### Determinations of SOD, CAT and APX activities

Frozen tissue powder (4 g) from 15 shoots was finely ground in liquid nitrogen and then homogenized in 20 ml of 0.05 M potassium phosphate buffer (pH 7.8) for SOD activity and 20 ml of 0.1 M potassium phosphate buffer (pH 7.0) for CAT and APX activities, respectively. The homogenate was filtered through two layers of miracloth and centrifuged at 20000 × g for 20 min at 4°C. The resulting supernatants were collected for the enzyme assays described below.

SOD activity was determined on the basis of the inhibition of nitroblue tetrazolium reduction to the blue formazan by superoxide radicals [25]. The specific SOD activity was expressed as unit (U)/mg protein.

CAT activity was assayed by measuring the reduction of H_2_O_2_ according to Change and Maehly [26]. One unit of CAT activity was defined as the amount of the enzyme that caused a change of 0.001 in absorbance per minute and then expressed as U/mg protein.

APX activity was measured following the oxidation of ascorbic acid at 290 nm (extinction coefficient 2.8 mM/cm) as per Nakano and Asada [27]. APX activity was defined as 1 μM ascorbate oxidized per minute at 290 nm and then expressed as U/mg protein.

### Determinations of lipase, PLD and LOX activities

Lipase was exacted from 4 g frozen tissue powder from 15 shoots with 20 ml of 0.2 M phosphate buffer (pH 7.8) containing 0.05 M mercaptoethanol for 1 min at 4°C [28]. One unit of enzyme activity was defined as the amount that caused a change of 0.001 in absorbance per minute. The specific lipase activity was expressed as U/mg protein.

PLD activity was measured by choline reinecke salt precipitation method described by Suttle and Kends [29]. Frozen tissue powder (4 g) from 15 shoots was homogenized in 20 ml of 0.1 M sodium acetate buffer (pH 5.6). The homogenate was centrifuged at 10000 × g and 4°C for 20 min and the supernatant was used for assaying PLD activity. One unit of PLD activity was defined as a change of 0.001 in absorbance at 520 nm per hour. The specific PLD activity was expressed as U/mg protein.

For analysis of LOX activity, 4 g of frozen tissue powder from 15 shoots was homogenized in 20 ml of phosphate buffer (pH 7.0). LOX activity was assayed at 25°C by monitoring the formation of conjugated dienes from linoleic acid at 234 nm according to the method of Axelrod et al. [30] and one unit of the LOX activity was defined as a change of 0.01 in absorbance per minute at 25°C. The specific LOX activity was expressed as U/mg protein.

### Determinations of ATP, ADP and AMP concentration

Extract and assays of ATP, ADP and AMP were conducted as per Liu et al. [31], with minor modifications. 2 g of frozen tissue powder from 15 WBS shoots was ground finely and homogenized with 7 ml of 0.6 M perchloric acid for 1 min in an ice bath. The extraction mixture was centrifuged at 6000 × g for 10 min at 4°C. 3 ml of supernatant was taken and quickly adjusted to pH 6.5−6.8 with 1 M potassium hydroxide (KOH), diluted to 5 ml and passed through 0.45 μm filter. ATP, ADP and AMP concentrations were determined using a high performance liquid chromatograph (Agilent-1200, Agilent, USA) equipped with a C_18_ reverse-phase column (Eclipse XDB-C_18_, 4.6 × 250 mm) and an ultraviolet (UV) detector at 254 nm. Mobile phase consisted of 0.06 M dipotassium hydrogen phosphate and 0.04 M potassium dihydrogen phosphate dissolved in deionized water and adjusted to pH 7.0 with 0.1 M KOH. The flow rate was 1.0 ml/min. Sample aliquots of 20 μl were injected into the HPLC. ATP, ADP and AMP concentrations were calculated according to the external standard programme and expressed as on fresh weight basis. EC was calculated as [ATP + 0.5 × ADP] × 100/[ATP + ADP + AMP].

### Determinations of protein content

Protein content was determined according to the method of Bradford [32], with bovine serum albumin used as the standard.

### Statistical analysis

All experiments were arranged in a randomized complete block design and the data were expressed as the means ± standard errors (SEs) from three replicates. Analysis of Variance was calculated using SPSS 10.0.

## Conclusions

The results demonstrated that modified atmosphere packaging in combination with low temperature storage maintained energy availability, increased antioxidant activity and decreased ROS production. In turn, this helped to decrease lipid degradation, maintain membrane integrity and then delay lignification and improve the sensory quality of minimally processed WBS.

## Competing interests

The authors declare that they have no competing interests.

## Authors’ contributions

LS made a significant contribution to acquisition of data, analysis and manuscript preparation. HC has made a substantial contribution to experimental design and data analysis. HG participated in study design and manuscript revision. XF participated in partial experiments. HM participated in partial experiments. YY participated in partial experiments and data analysis. QY participated in partial experiments. YJ made a significant contribution to experimental design and manuscript revision. All authors read and approved the final manuscript.
